# Detection and Reconstruction of Passion Fruit Branches via CNN and Bidirectional Sector Search

**DOI:** 10.34133/plantphenomics.0088

**Published:** 2023-09-08

**Authors:** Jiangchuan Bao, Guo Li, Haolan Mo, Tingting Qian, Ming Chen, Shenglian Lu

**Affiliations:** ^1^ Key Lab of Education Blockchain and Intelligent Technology, Ministry of Education, Guangxi Normal University, Guilin 541004, China.; ^2^ Guilin Center for Agricultural Science & Technology Research, Guilin 541006, China.; ^3^ Agricultural Information Institutes of Science and Technology, Shanghai Academy of Agriculture Sciences, Shanghai 201403, China.; ^4^ Guangxi Key Lab of Multi-Source Information Mining and Security, Guangxi Normal University, Guilin, 541004, China.

## Abstract

Accurate detection and reconstruction of branches aid the accuracy of harvesting robots and extraction of plant phenotypic information. However, the complex orchard background and twisting growing branches of vine fruit trees make this challenging. To solve these problems, this study adopted a Mask Region-based convolutional neural network (Mask R-CNN) architecture incorporating deformable convolution to segment branches in complex backgrounds. Based on the growth posture, a branch reconstruction algorithm with bidirectional sector search was proposed to adaptively reconstruct the segmented branches obtained by an improved model. The average precision, average recall, and F1 scores of the improved Mask R-CNN model for passion fruit branch detection were found to be 64.30%, 76.51%, and 69.88%, respectively, and the average running time on the test dataset was 0.75 s per image, which is better than the compared model. We randomly selected 40 images from the test dataset to evaluate the branch reconstruction. The branch reconstruction accuracy, average error, average relative error of reconstructed diameter, and mean intersection-over-union (mIoU) were 88.83%, 1.98 px, 7.98, and 83.44%, respectively. The average reconstruction time for a single image was 0.38 s. This would promise the proposed method to detect and reconstruct plant branches under complex orchard backgrounds.

## Introduction

Traditional fruit production operations are highly labor-intensive, with labor costs accounting for more than half of the total cost [1], which makes the shortage of skilled labor and increasing costs challenging for agriculture [2]. With the widespread use of information technology, agricultural automation has been studied extensively [3]. Intelligent agricultural robots with strong perception and reasoning capabilities are applied to tedious and repetitive agricultural production work to solve problems such as labor shortages [4]. However, in the unstructured orchard environment, irregularly growing plant branches often hinder the operations of automated robots. Therefore, accurate detection and reconstruction of plant branches in natural environments are necessary prerequisites for achieving automation and intelligence in operations such as fruit picking and branch pruning, while also providing necessary perceptual technology support for the production operation of other intelligent agricultural equipment and facilitating the extraction of plant phenotype information.

In earlier studies, the detection and reconstruction of plant branches were mainly based on traditional vision techniques, including color space transformation, edge detection, thresholding, and cluster segmentation. In the work of Ji et al. and He et al. [5,6], fruit branches were extracted and segmented by adjusting the color parameters of the images, calculating grayscale differences, and combining edge information. However, complete branch information could not be obtained because the segmented branches were discontinuous. Tabb and Medeiros [7] use super-pixel to determine the background of low-texture regions and combined with color distribution to segment branches. Juman et al. [8] combine the Viola and Jones detectors with a preprocessing method using color space information to segment the trunk. Although these methods can extract branches from digital images, their backgrounds are relatively simple. In a natural orchard environment, these methods are subject to various complications that can lead to a decline in recognition accuracy. Another disadvantage of digital image detection is that a single image has only one viewing angle, which is easily affected by occlusion. Therefore, detecting and reconstructing branches from 3-dimensional information has become a popular approach. For example, You et al. [9] proposed an algorithm to model the branch skeleton using a label-dependent topology and geometric priors, and they achieved a median accuracy of 70%. Ma et al. [10] used 2 synchronous consumer-level RGB-D cameras to construct a vision system and used SPGNet to segment point cloud data of a jujube tree; they achieved 0.84 branch accuracy and 0.76 branch intersection-over-union (IoU). Westling et al. [11] extracted and fitted branching curves from point-cloud data based on graph operations. The main disadvantage of these methods is that they need to obtain high-quality point clouds. Although both of these methods have made progress, they are easily influenced by complex factors in natural orchard environments such as lighting and wind. Subsequent research needs to further reduce equipment costs and dependence on the environment while improving the accuracy of branch detection and reconstruction, in order to achieve branch reconstruction in actual orchards.

Recently, researchers have begun to apply deep learning to the detection of plant branches. Compared with the above traditional methods, the powerful feature extraction and autonomous learning abilities of deep neural networks can better adapt to complex backgrounds [12–14]. In 2 studies [15,16], SegNet was used to segment the trunk and branches of apple trees with common appearance and features, and IoU scores of 0.59 and 0.44 and boundary F1 scores of 0.93 and 0.89 were obtained. In 2 other studies [17,18], different convolutional neural network (CNN) architectures were used to detect and segment apple tree branches and achieved improved performance. Yang et al. [19] used Mask R-CNN to segment citrus branches and proposed a multi-constraint method to reconstruct branches, which achieved an 88.64% reconstruction accuracy rate. Wan et al. [20] used a CNN to roughly segment branches and combined it with image processing and polynomial fitting methods to reconstruct branches accurately, finally obtaining a reconstruction accuracy of 88.76%. The aforementioned studies all focused on arborvitae fruit trees, and there was an obvious contrast between the objectives and background, which is relatively simple. The parameters that constrain branch reconstruction must be obtained by a large amount of data analysis in the early stage, which makes the entire method inflexible.

**Fig. 1.**
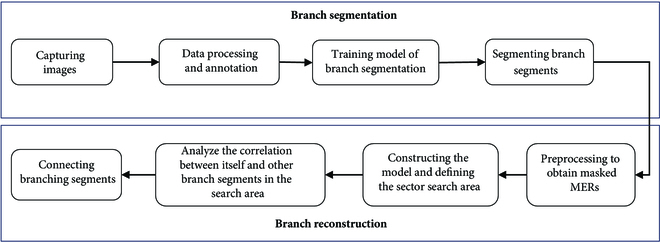
Overview of the proposed method for reconstructing passion fruit branches.

Therefore, the objectives of this study were as follows: (a) to develop an improved instance segmentation network to accurately detect and segment curved and irregular branches of vine-like fruit tree under natural orchard environment, and (b) to design a branch reconstruction algorithm that is expected to adaptively reconstruct branches from the above detected branch segments to obtain complete branch information. Figure 1 presents the overview of the proposed method.

## Materials and Methods

### Data acquisition and annotation

**Fig. 2.**
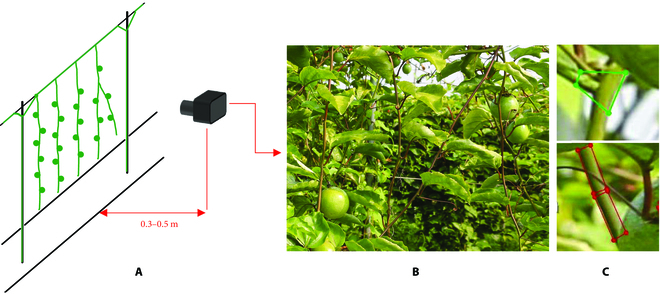
Image acquisition and annotation: (A) camera position and angle, (B) examples of captured images, and (C) labels of the forked (top) and segmented (bottom) branches.

The golden passion fruit tree images used in this study were captured in September 2021 from an orchard located in Liutang Town, Guilin City, Guangxi Province, China. An Intel RealSense D455 depth camera was used to capture images with a resolution of 1,280 × 720 pixels. As Fig. 2A shows, the distance between the camera and branches was approximately 0.3 to 0.5 m. To achieve diversity of samples, these images were captured at 3 different time points: 0900, 1400, and 1600. In total, 133 images were captured. These original images were augmented to 1,742 images by using data augmentation techniques (add noise, add blur, mirror flip, rotate, adjust image brightness, etc.), and approximately 90,000 annotated instances were obtained. The training and test sets were divided in a ratio of 7:3.

In the orchard environment, the growth posture of passion fruit branches is random; to better identify and reconstruct the branches, in this study, we adopted the labeling method proposed by Yang et al. [19] to represent randomly growing branches with regular-shaped quadrilaterals, which effectively reduces the complexity of detecting the original branches. Figure 2C shows the effect of image annotation. To better represent a branch, the aspect ratio of the quadrilateral was set in the range of 2 to 4, and a smaller quadrilateral mark was used for branches with a larger curvature.

### Improved mask R-CNN for branch detection and segmentation

**Fig. 3.**
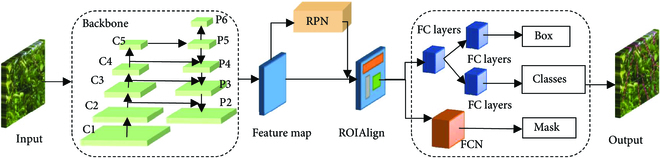
Structure diagram of Mask R-CNN.

In this study, Mask R-CNN [21] was used to segment the passion fruit branches. Compared with other detection models, the masks predicted by Mask R-CNN can more accurately represent branch information. Mask R-CNN comprises a backbone network, a region proposal network (RPN) [22], and a head network. The backbone uses ResNet101 [23] and a feature pyramid network (FPN) [24] to extract and fuse features. In Fig. 3, the input of ResNet101 is an RGB image, and the output is a feature map of different stages, from low to high. The feature map output by the high stage contains high-level semantic information such as shape, location, category, etc., and the feature map output by the low stage mainly contains low-level feature information, such as color, texture, and edges. The feature maps generated at each stage are input into the FPN to obtain stronger semantic information. Then, the region of interest (ROI) is generated on the feature map through the RPN, and each ROI is input into the head network. The head network is divided into 2 branches: detector and mask. The detector branch is used to predict the category and bounding box of the object, and the mask branch is used to predict the binary mask of the object.

**Fig. 4.**
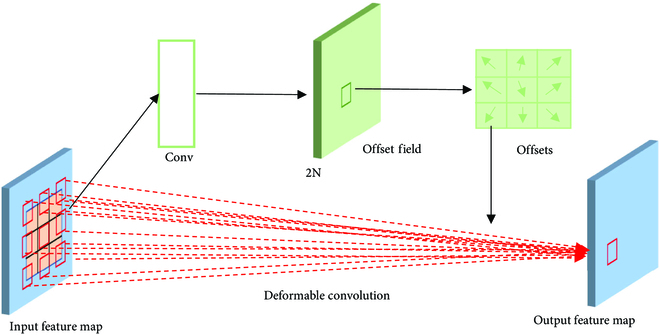
Architecture of a 3 × 3 deformable convolution.

The objects detected and segmented in this study are curved and irregular passion fruit branch, and the objects are similar in color to the background; the study made some improvements to ResNet so that the model can segment objects more accurately. Considering the poor adaptability of ordinary convolution to unknown changes and its weak generalization ability, it cannot perform better feature extraction for plant branches with different scales and deflection angles. This study integrated the deformable convolution proposed in Dai et al. [25,26] into ResNet. As shown in Fig. 4, the receptive field can be automatically adjusted to fit the shape of the object by adding an offset to each sample point in the convolution kernel. The offset calculation is a parallel process. The deviation of the convolution kernel is learned by adding a parallel standard convolution, and an offset is added to the convolution kernel such that the sampling point position of the convolution kernel on the feature map changes with adaptive changes in the image content, thereby improving the generalization ability of objects with different shapes.

**Fig. 5.**
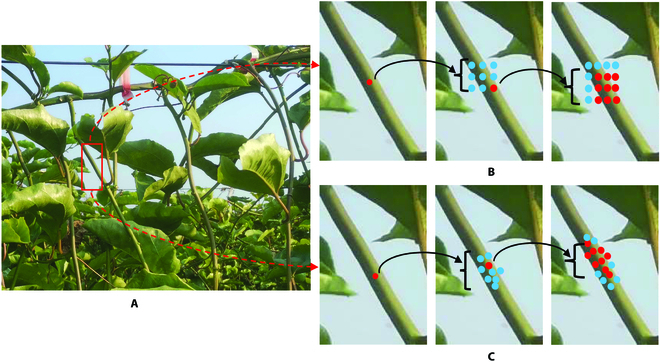
The comparison of the sampling process: (A) RGB image, (B) the sampling process of ordinary convolution, and (C) the sampling process of deformable convolution.

The comparison of the sampling process between ordinary convolution and deformable convolution in this paper is shown in Fig. 5. In Fig. 5B and C, 2 activation points are first selected on the branch, and preliminary sampling points are obtained after applying a 3 × 3 convolution. Then, another 3 × 3 convolution was applied to obtain the final sampling points. By comparing the two, it can be observed that the sampling points with added offsets were no longer in a square shape, which made the sampling positions of deformable convolution more aligned with the shape and size of the branch itself compared to regular convolution.

### Branch reconstruction

#### Algorithm description and postprocessing

**Fig. 6.**
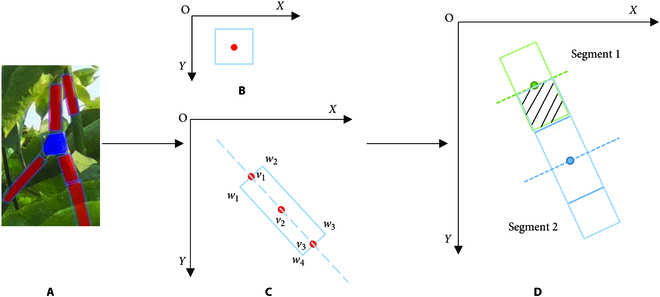
Simplified branch segment: (A) branch segment after minimum bounding rectangle processing, (B) simplified forked branch, (C) simplified segmented branch, and (D) critical case satisfying the minimum IoU of the reconstruction algorithm.

After the branch image is processed by the improved Mask R-CNN, the bounding box, category, and binary mask information of the branch are obtained. To obtain complete branch information, this paper proposes a branch reconstruction algorithm based on a bidirectional sector search that uses the binary mask information of the object. The minimum bounding rectangle containing all pixels of the extracted mask is calculated, and the segmented branch is replaced with the minimum bounding rectangle for subsequent branch reconstruction to reduce the influence of an incomplete mask on the branch reconstruction accuracy. Figure 6A shows the effect of the extraction, where the red and blue regions are the extracted masks, and the blue box in Fig. 6B and C is the calculated minimum bounding rectangle, where {*v*_1_, *v*_2_, *v*_3_} represent the set of points on the long median axis of the segmented branch. {*w*_1_, *w*_2_, *w*_3_, *w*_4_} represent the 4 corner points of the minimum bounding rectangle starting from the top left corner clockwise.

The branch mask generated after the input image is segmented by the model, which sometimes causes duplication. In this case, the branch reconstruction algorithm causes a branch reconstruction error. To reduce the occurrence of this type of error, calculate the IoU between the extracted minimum bounding rectangles and discard the minimum bounding rectangles whose IoU is greater than the threshold. In the image labeling stage, set the quadrilateral aspect ratio of the label to be in the range of 2 to 4; therefore, the aspect ratio of the minimum bounding rectangle for extracting the segmentation mask is also approximately in the range of 2 to 4. Figure 6D shows a critical case that satisfies the minimum IoU of the reconstruction algorithm. The intersection ratio was set to 0.2. Considering the deviation in the short-axis direction of the minimum bounding rectangle, the threshold was set to 0.15. The preprocessed segmented branches were used to reconstruct the last complete branch using a reconstruction algorithm based on a bidirectional sector search. The specific steps are presented in Algorithm 1.


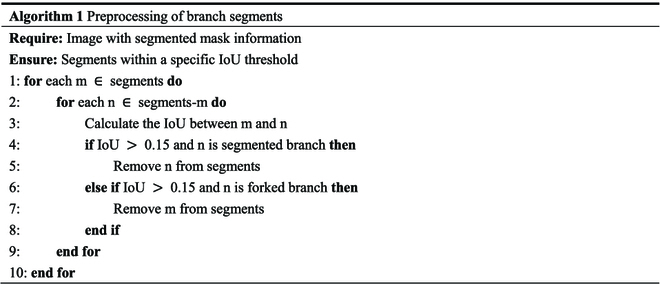


#### Segmented branches reconstruction based on bidirectional sector search

According to the growth posture of passion fruit branches, segmented branches belonging to the same branch are always distributed according to a certain growth direction. However, owing to the problems of occlusion and excessive bending of passion fruit branches, the relative angles of segmented branches from the same branch have different degrees of deviation. The method proposed by Amatya et al. [27] misses branch segments belonging to the same branch. Therefore, a sector search area was constructed to search as many branch segments of the same branch as possible. To better fit the branches and adapt to the zigzag growth posture of the branches, for the branching part, this paper simplifies the minimum bounding rectangle of the preprocessed mask with a segmented branch model *B* = (*V*, *W*), where *V* = {*v*_1_, *v*_2_, …, *v_n_*} denotes the set of points on the long median axis of the segmented branch; for the initial segmented branch, *V* contains only 3 points. *W* = {*w*_1_, *w*_2_, *w*_3_, *w*_4_} denotes the 4 corner points of the minimum bounding rectangle starting from the top left corner, moving clockwise, which is used to determine the sector search boundary. Use the center point of the minimum bounding rectangle to represent the bifurcation part. The simplified branch mask is shown in Fig. 6B and C.

**Fig. 7.**
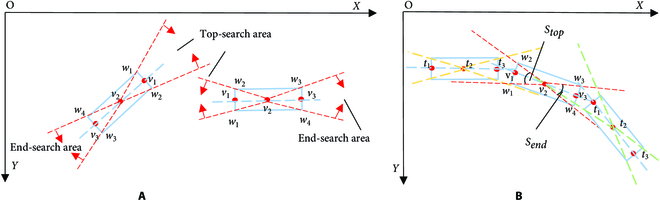
Initial reconstruction flowchart: (A) defining the search area and (B) search process of the branch segment.

Branches always grow at both ends (above and below or left and right) relative to the current segmented branch. Therefore, for each segmented branch, a bidirectional sector search area is constructed along its long axis based on the simplified branch model, where the top search area is assumed to be above and left of the segmented branch, and the end search area is assumed to be below and right of the segmented branch. In Fig. 7A, dashed lines *v*_2_*w*_1_ and *v*_2_*w*_2_ limit the boundary of the top-sector search area, and dashed lines *v*_*n* − 1_*w*_3_ and *v*_*n* − 1_*w*_4_ limit the boundary of the end-sector search area (*n* = 3). All other segmented branches in the image that lie within the target segmented branch search area are searched. Define the sector angles *S_top_* and *S_end_* of the search area and offset angles *D_top_* and *D_end_* of the points constituting other segmented branches relative to the target segmented branch, as shown in Eqs. 1 and 2.

$$\left\{\begin{array}{c} S_{\mathrm{top}}=\arccos\frac{\vec{v_{2}w_{1}}\cdot \vec{v_{2}w_{2}}}{\left|\vec{v_{2}w_{1}}\right|\left|\vec{v_{2}w_{2}}\right|}\\ D_{\mathrm{top}}=\arccos\frac{\vec{v_{2}t_{p}}\cdot \vec{v_{2}v_{1}}}{\left|\vec{v_{2}t_{p}}\right|\left|\vec{v_{2}v_{1}}\right|} \end{array}\right.$$ (1)

$$\left\{\begin{array}{c} S_{\mathrm{end}}=\arccos\frac{\vec{v_{n-1}w_{3}}\cdot \vec{v_{n-1}w_{4}}}{\left|\vec{v_{n-1}w_{3}}\right|\left|\vec{v_{n-1}w_{4}}\right|}\\ \;D_{\mathrm{end}}=\arccos\frac{\vec{v_{n-1}t_{p}}\cdot \vec{v_{n-1}v_{n}}}{\left|\vec{v_{n-1}t_{p}}\right|\left|\vec{v_{n-1}v_{n}}\right|} \end{array}\right.$$ (2)

where *t_p_*(*p* ∈ {1,2,…, *n*}) represents the long axis points that constitute other branch segment models, and *n* represents the total number of point sets of the target branch segment model. When $D_{\mathrm{top}}\leq \frac{1}{2}S_{\mathrm{top}}\;\left(p=n\right)$, the segmented branch is temporarily marked in the top search area of the target branch segment. When $D_{\mathrm{end}}\leq \frac{1}{2}S_{\mathrm{end}}\;\left(p=1\right)$, the branch segment is temporarily marked in the end search area of the target branch segment.

$$\left\{\begin{array}{c} O_{\mathrm{top}}=\arccos\frac{\vec{v_{2}v_{1}}\cdot \vec{t_{n}t_{n-1}}}{\left|\vec{v_{2}v_{1}}\right|\left|\vec{t_{n}t_{n-1}}\right|}\\ O_{\mathrm{end}}=\arccos\frac{\vec{v_{n-1}v_{n}}\cdot \vec{t_{1}t_{2}}}{\left|\vec{v_{n-1}v_{n}}\right|\left|\vec{t_{1}t_{2}}\right|} \end{array}\right.$$ (3)

To divide the branch segments more accurately and reduce the influence of adjacent branches, the strategy of searching the target branch segment in reverse is adopted for the marked branch segment, and Eq. 3 is used to constrain the offset angle of the two to further ensure that it is on the same branch as the target branch, i.e., $O_{\mathrm{top}}\leq \frac{1}{2}S_{\mathrm{top}}$ or $O_{\mathrm{end}}\leq \frac{1}{2}S_{\mathrm{end}}$. When the simplified center point of a forked branch falls within the search area, it is considered to be on the same branch as the target branch segment. Figure 7B shows the branch segment search process.

$$\left\{\begin{array}{c} {\mathrm{dis}}_{\mathrm{top}}=\text{distance}\left(v_{1},v_{3}\right)\\ {\mathrm{dis}}_{\mathrm{end}}=\text{distance}\left(v_{n-2},v_{n}\right) \end{array}\right.$$ (4)

Equation 4 constrains the depth of the search range. Considering the problems of occlusion and leakage identification, after numerous experiments, set the depth of the search-segmented and forked branches to 3*dis*_top_/3*dis*_end_ and 1.6*dis*_top_/1.6*dis*_end_, respectively. When calculating the distance between the target branch segment and other segmented and forked branches in the search area, if the closest distance is a forked branch, it is merged with the target branch segment, and the search for connections ends. If the closest distance is a segmented branch, it is merged with the target branch segment, the target branch segment is updated, and the search process is repeated for the updated target branch segment until it is connected to a forked branch or there are no other branch segments belonging to the same branch in the search area. The specific steps are presented in Algorithm 2.


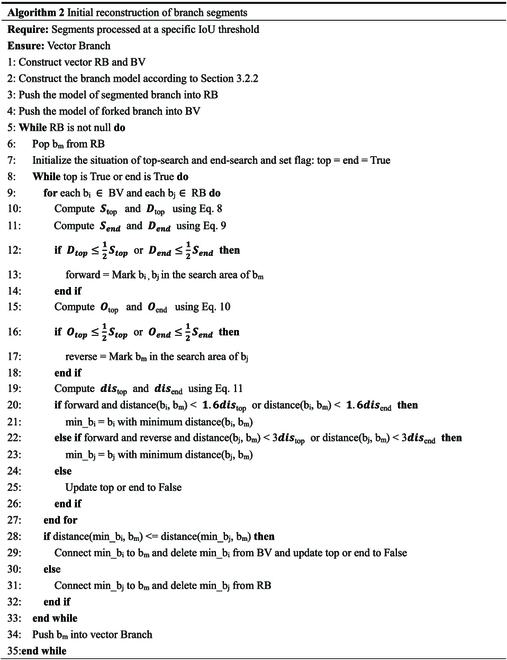


#### Secondary connection of branch segments from the same branch

**Fig. 8.**
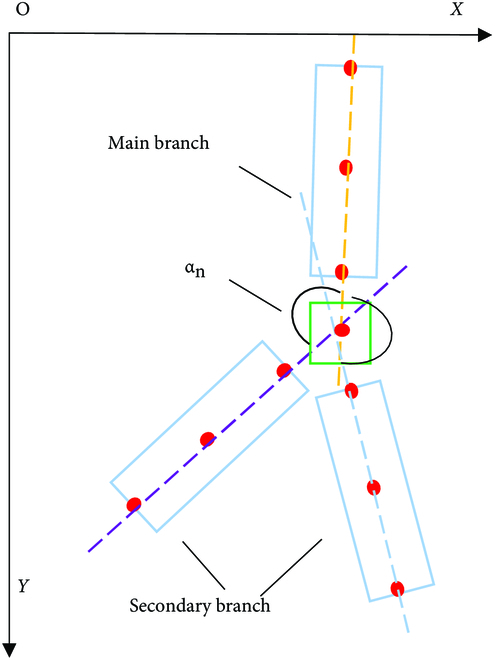
Multiple branches with the same bifurcation point.

**Fig. 9.**
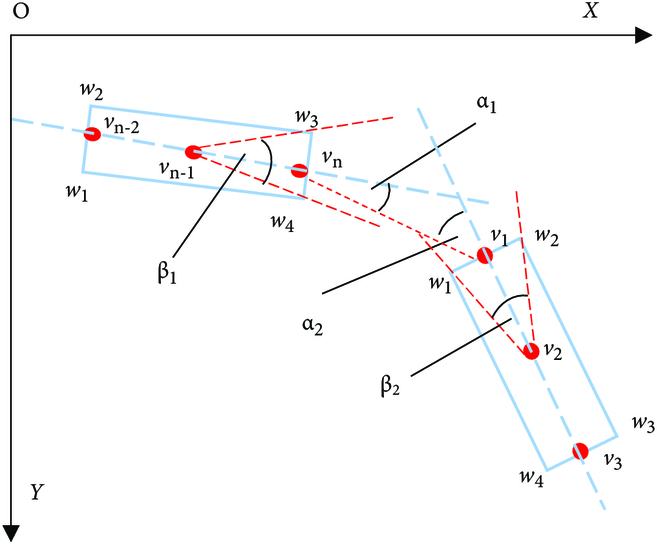
Constraints on deflection angle.

After reconstruction using the bidirectional sector search reconstruction method described in the “Segmented branches reconstruction based on bidirectional sector search” section, the individual segmented and forked branches are connected to the same branch. However, because of the problems of forked lateral branches, missed detection, and occlusion, the same complete branch will be divided into multiple segments when connecting. Therefore, secondary connections are made to other branch segments belonging to the same branch. For multiple branches with the same bifurcation point, the branch whose bifurcation point is the tail node is defined as the main branch, and the branch whose bifurcation point is the top node is the second branch, as shown in Fig. 8. Due to missed detection, there may only be a pair of main branches and secondary branches that do not belong to the same branch, so a threshold is set to constrain the secondary branches, and the threshold is set to 100° considering the critical situation, i.e., when the primary branch is perpendicular to the secondary branch and when the branches are bent to different degrees. Thus, the relative angle *α_n_*(*n* = 1, 2) between the main branch and each secondary branch is calculated, and if max(*α_n_*) > 100^°^, then it is connected with the main branch. For the multi-segment branches that do not have the same bifurcation point, the search and connection method introduced in the “Segmented branches reconstruction based on bidirectional sector search” section is used. At this time, the angle of the sector search area is constrained within the range of *D_top_* ≤ *S_top_* (*p* = *n*) and *D_end_* ≤ *S_end_* (*p* = 1), and the distance is constrained within the range of 5*dis*_top_ or 5*dis*_end_. The constraints shown in Fig. 9(*α*_1_ ≤ *β*_1_ and *α*_2_ ≤ *β*_2_) were applied to the deflection angles to reduce the influence of parallel branches on the reconstruction. Branch detection and segmentation are not always correct; it could also wrongly detect leaves and petioles. Therefore, in the final reconstruction results, our algorithm deletes the individual segmented branch (in most cases, leaves and petioles do not meet the reconstruction requirements; it is required to still maintain the individual segmented branch) to reduce the reconstruction error.

### Evaluation metrics

This study verified and evaluated the effectiveness of the proposed method in terms of branch segmentation and reconstruction.

1. To compare the detection and segmentation performances of the improved Mask R-CNN and other networks on the test set, the precision, recall rate, and F1 of the model were calculated as follows:

$$\text{precision}=\frac{\mathrm{TP}}{\mathrm{TP}+\mathrm{FP}}$$ (5)

$$\text{recall}=\frac{\mathrm{TP}}{\mathrm{TP}+\mathrm{FN}}$$ (6)

$$F_{1}=\frac{2\times P\times R}{P+R}$$ (7)

where *TP* is true positive, *FN* is false negative, and *FP* is false positive.

2. To verify the reconstruction effectiveness of the algorithm on passion fruit branches, we used *Acc_r_* to evaluate the overall reconstruction effect of the algorithm and *mIoU* to evaluate the fitting effect between the reconstructed and real branches as follows:

$$\mathrm{Ac}c_{r}=\frac{R_{b}}{G_{b}}\times 100\%$$ (8)

where *Acc_r_* is the reconstruction accuracy, *R_b_* is the number of correctly reconstructed branches, and *G_b_* is the actual number of branches, and

$$\text{mIoU}=\frac{\sum_{i=1}^{N}\left(\frac{R_{i}\cap G_{i}}{R_{i}\cup G_{i}}\right)}{N}\times 100\%$$ (9)

where *mIoU* is the average reconstructed IoU, *R_i_* is the pixel value of the reconstructed branch, *G_i_* is the actual branch pixel value, and *N* is the number of branches in the experimental group.

3. The average error and average relative error are defined to evaluate the estimation performance of the branch size as follows:

$$E_{a}=\frac{\sum_{n=1}^{N}\;\left|D_{\mathrm{rn}}-D_{\mathrm{gn}}\right|}{N}$$ (10)

$$E_{r}=\sum_{n=1}^{N}\frac{\left|D_{\mathrm{rn}}-D_{\mathrm{gn}}\right|}{{\mathrm{ND}}_{\mathrm{gn}}}\times 100\%$$ (11)

where *E_a_* and *E_r_* are the mean errors and mean relative errors of the branch diameters, *D_rn_* is the branch diameter obtained by the proposed method, *D_gn_* is the measured diameter of passion fruit branches, and *N* is the number of data used in the experiment.

## Results and Discussion

To evaluate the effectiveness of the proposed branch identification and reconstruction algorithm, the following experiments were designed, and the results were analyzed. The computer configuration used in the experiment was as follows: Intel Xeon Gold 6230 CPU, NVIDIA Tesla V100 GPU, and Ubuntu 18.04 operating system. The branch segmentation models (Mask R-CNN, BlendMask [28], and Cascade Mask R-CNN [29]) used in the experiment were all implemented based on the Detectron2 platform. The parameter settings are recommended by the official documents, with modifications on some parameters as follows: workers: 2, batch size: 4, epoch: 100, learning rate: 0.001.

### Analysis of branch segmentation results

The comparative experiments for evaluating the segmentation performance of our improved Mask R-CNN were conducted with BlendMask, Cascade Mask R-CNN, and the original Mask R-CNN, on metrics precision, recall, and F1 scores. The results are listed in Table 1. We can see that the improved Mask R-CNN achieved the best results on our test dataset of passion fruit tree. Compared with the original Mask R-CNN, the precision, recall, and F1 scores of the improved model on "Bbox" were 4.28%, 2.95%, and 3.77% higher, respectively, and on "Segm", they were 4.52%, 3.15%, and 4.04% higher, respectively. Compared with the original network, our improved model achieves obvious improvement in these evaluation metrics. This proves that, in this case, the improved model can better detect and segment passion fruit branches. However, the overall detection performance of the improved model is still not sufficient. This may because (a) relatively few data were used for training in our model; (b) some branches were correctly identified and segmented but were inconsistent with the manually labeled ground-truth branches; and (c) the color of some branches is similar to the background color (leaves and fruits), and the detection model cannot extract the feature information of the branches well, resulting in some missed detections and false segmentations.

**Table 1.** Comparison results among several segmentation networks.

Methods	Bbox (%)			Segm (%)		
	Precision	Recall	F1	Precision	Recall	F1
BlendMask	52.27	71.39	60.35	47.96	67.53	56.09
Cascade Mask R-CNN	58.48	74.63	65.58	51.86	68.43	59.00
Mask R-CNN	60.02	73.56	66.11	52.61	66.75	58.84
Improved Mask R-CNN	**64.30**	**76.51**	**69.88**	**57.13**	**69.90**	**62.88**

**Fig. 10.**
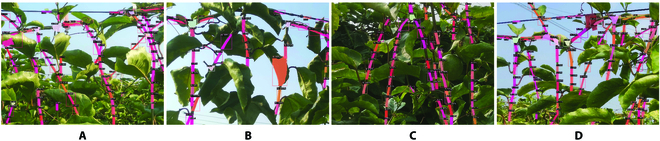
Detection and segmentation results of branches: (A) detected result with some errors on forked branches, (B) detected result with some errors from leaves, (C) detected result with missing detection on branches, and (D) detected result with some errors from wire rope.

Figure 10 shows the visual results of branches’ detection and segmentation from our improved Mask R-CNN model. We can see that the improved Mask R-CNN can detect and segment accurately most of the passion fruit branches with a complex background under a natural environment. However, we can also find that few leaves and petioles were wrongly detected.

**Fig. 11.**
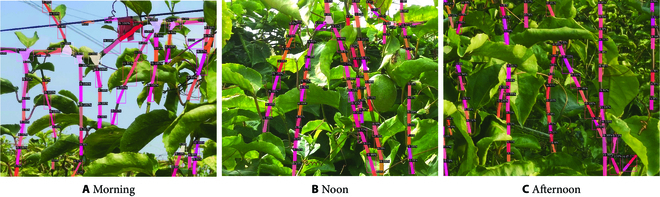
Improved Mask R-CNN detection effect under 3 sunlight conditions: (A) detected result from an image captured in the morning, (B) detected result from an image captured at noon, and (C) detected result from an image captured in the afternoon.

To verify the robustness of our improved Mask R-CNN, comparative experiments were carried out on these comparative models under 3 sunlight conditions, and the experimental results are shown in Table 2. Compared with other models, our improved Mask R-CNN has better performance indicators under 3 sunlight conditions. In order to demonstrate the effects of the improved model more intuitively, Fig. 11 shows the visual detected results.

**Table 2.** Model comparison using the test set under 3 sunlight conditions.

Methods	Sunlight condition	Bbox (%)			Segm (%)		
		Precision	Recall	F1	Precision	Recall	F1
BlendMask	Morning	55.71	75.93	64.26	46.63	68.36	55.44
Cascade Mask R-CNN		65.63	78.59	71.53	57.86	71.91	64.12
Mask R-CNN		66.15	77.85	71.53	57.32	69.98	63.02
Improved Mask R-CNN		**70.02**	**79.76**	**74.57**	**58.33**	**70.06**	**63.66**
BlendMask	Noon	55.11	71.65	62.30	49.45	66.93	56.88
Cascade Mask R-CNN		62.51	76.76	68.91	53.40	68.63	60.07
Mask R-CNN		64.52	76.48	70.00	54.34	67.98	60.40
Improved Mask R-CNN		**67.37**	**78.17**	**72.37**	**56.87**	**69.65**	**62.62**
BlendMask	Afternoon	51.00	72.48	59.87	47.40	68.32	55.96
Cascade Mask R-CNN		59.79	76.11	66.97	54.33	71.07	61.58
Mask R-CNN		60.67	75.45	67.26	54.81	69.93	61.45
Improved Mask R-CNN		**66.36**	**78.83**	**72.06**	**59.21**	**72.80**	**65.31**

### Evaluation of branch reconstruction

**Fig. 12.**
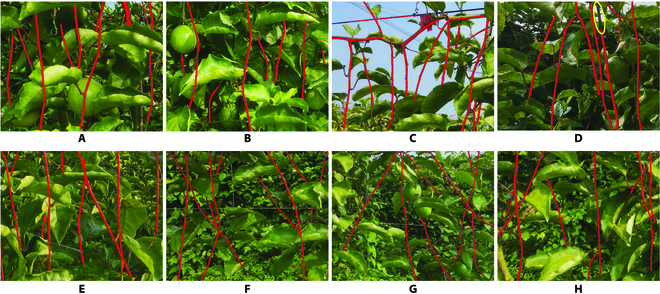
Results of branch reconstruction under complex environmental conditions: (A to D) reconstructed results on different lighting and background conditions and (E to H) reconstructed results on different occlusion.

**Fig. 13.**
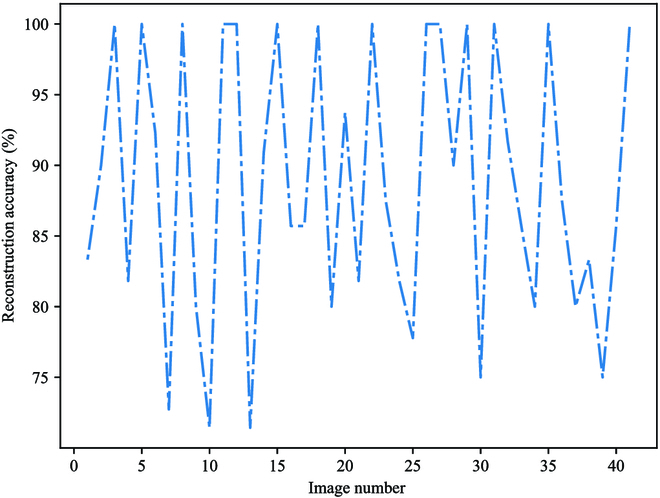
Reconstruction accuracy on different images.

**Fig. 14.**
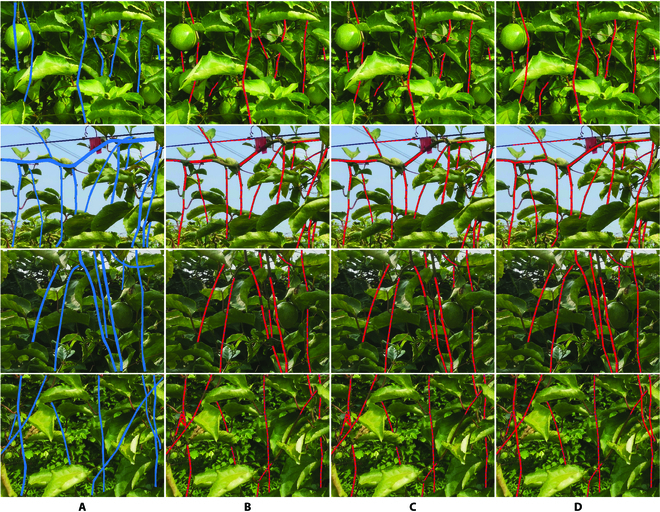
The branch reconstructed effects of 3 methods on passion fruit tree. (A) The ground-truth branches, (B) the reconstructed results by using the method presented in Yang et al. [19], (C) the reconstructed results by using the method presented in Wan et al. [20], and (D) the reconstructed results of our model.

To verify the performance of our proposed algorithm for branch reconstruction, 40 images were randomly selected. Figure 12 shows the visual reconstructed results under different environmental conditions. Because of the segmentation error from the segmentation model, the 2 ends of the reconstructed branches could not completely fit the length of the actual branches, as shown in Fig. 12D. Therefore, the number of correctly reconstructed branches was counted for each reconstructed image. Figure 13 shows the distribution of reconstruction accuracy of our improved model on these test images, in which the reconstructed accuracy for each image was calculated by using Eq. 8, where the ground-truth branches were labeled manually on the image, as shown in Fig. 14A. In this study, a reconstructed branch can be counted as a correctly reconstructed branch if the IoU of this reconstructed branch with the manual labeled ground-truth branch is greater than 0.9. This can be calculated by pixel coverage on the 2 images.

Another experiment was conducted to compare the performance of branch reconstruction of our method with the methods proposed by Yang et al. [19] and Wan et al. [20]. We reimplemented the 2 methods by using Python language in Windows 10, and reconstructed branches of passion fruit from the branch segments detected by using our improved Mask R-CNN. The experimental results are shown in Table 3, in which the reconstructed accuracy was calculated by averaging the reconstructed accuracy in each image on the test dataset. Table 3 shows that the reconstruction accuracy of our proposed model is much higher than that of the other 2 methods on this dataset of passion fruit tree. While the other 2 methods achieved significantly low reconstructed accuracy, this could be due to the fact that there are more complex occlusion in our dataset compared to their experimental environment.

**Table 3.** Accuracy comparison of different reconstruction methods.

Reconstructed method	Reconstructed accuracy (%)
Yang et al. [19]	48.19
Wan et al. [20]	47.44
Ours	88.83

Figure 14 shows the visual reconstruction effects of these 3 comparative methods. The manual labeled ground-truth branches are shown in Fig. 14A. It can be seen from this figure that our reconstruction method can better adapt to occlusion, missed detection, and other complex situations under a natural environment, and reconstruct more complete branches.

**Fig. 15.**
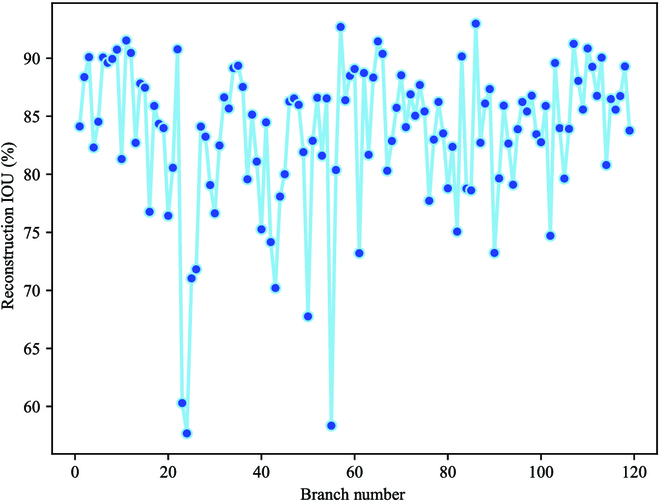
Distribution of reconstructed branch IoUs.

**Fig. 16.**
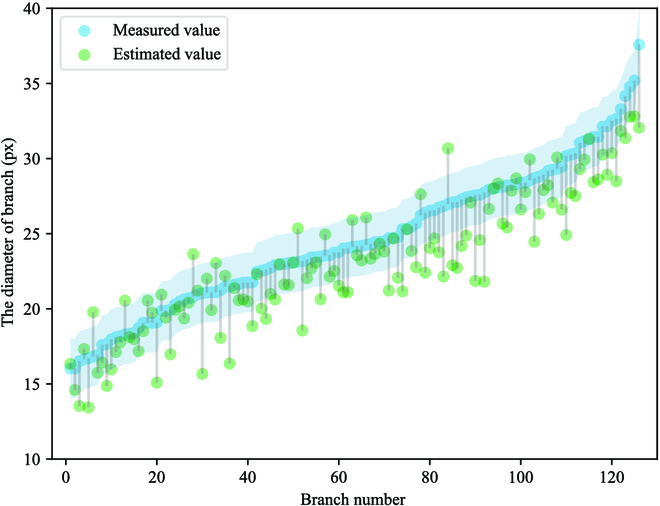
Distribution of deviations between measured and estimated diameters of branches.

In Fig. 14, the proposed method still showed good performance and slightly higher reconstruction accuracy than the other methods. However, some errors still existed, mainly owing to serious occlusion and excessively tortuous branches. To evaluate the effectiveness of our proposed method more accurately, we used Eqs. 9, 10, and 11 to calculate the *E_a_*, *E_r_*, and mean IoU (mIoU) of the reconstructed branch. The results are listed in Table 4, where the mIoU is 83.44%. Figure 15 shows the overall distribution of the branch IoU, and Fig. 16 shows the deviation between the measured and estimated diameters of the branches, where the light blue area is the confidence interval of the branch diameter in the range of 1.98 px.

**Table 4.** Performance of the proposed method in terms of *E_a_*, *E_r_*, and mIoU.

Evaluation parameter	Reconstructed branches
mIoU (%)	83.44
Average error of branch diameter (*E_a_*) (px)	1.98
Average relative error of branch diameter (*E_r_*) (%)	7.98

### Time efficiency analysis

The detection and reconstruction of branches are mainly used in agricultural engineering applications, such as obstacle avoidance by harvesting robots and detection of plant growth status. Therefore, time efficiency must be analyzed. Table 5 shows our experimental results on the test set, where the average running time and standard deviation for a single image were 1.13 s and 0.135 s, respectively. This satisfies the requirements of agricultural engineering applications.

**Table 5.** Average and standard deviation of running time per image.

Task category	Average time (s/image)	Standard deviation (s)
Branch segmentation	0.75	0.117
Branch reconstruction	0.38	0.018
Total	1.13	0.135

## Conclusion

This study designed a complete pipeline to detect and reconstruct branches of vine-like fruit trees. Deep learning methods were used to identify and segment branch regions in passion fruit tree images. Due to the excessive bending and irregular growth characteristics of passion fruit tree branches, deformable convolution was integrated into the feature extraction module of Mask R-CNN to enhance its detection and segmentation capabilities for irregularly shaped passion fruit tree branch segments. By comparing the detection and segmentation results of various models, it was found that the improved model could better detect and segment passion fruit tree branches with near-color backgrounds, overlap, and occlusion. The improved model achieved accuracy, recall, and F1 scores of 64.30%, 76.51%, and 69.88% for identification, as well as accuracy, recall, and F1 scores of 57.13%, 69.90%, and 62.88% for segmentation. The average segmentation time per image for this model was 0.75s.

Since it is not possible to extract complete branch information directly from the model's detection results, a branch reconstruction algorithm based on bidirectional sector search was proposed in this paper, which can adaptively reconstruct branches with minor parameter adjustments by combining sector search strategies on the output results of the detection model in some specific ways. It should be noted that the sector search strategy used in the reconstruction algorithm has a wider search area at longer distances, which partially compensates for low reconstruction rates due to excessive bending of branches or missed detections by the detection model to some extent. Experiments were conducted on 40 test images in this paper where branch reconstruction accuracy reached 88.83%. The mean errors in reconstructed branch diameter and relative error were 1.98 px and 7.98%, respectively; while the mIoU was 83.44%.

Although this method can improve overlapping, missing, or bent branches, there are still some problems that need to be addressed. Firstly, the detection model may still have problems such as missed detections or false positives, and its detection accuracy for particularly small branches is not satisfactory. A more effective detection model can be sought after and different optimization modules can be selected for specific issues to enhance detection speed while ensuring accuracy. Secondly, the branch reconstruction method was proposed specifically for passion fruit tree branches, so more types of fruit tree branches need to be collected to further optimize this method. Overall, the proposed method can provide a good foundation for detecting and extracting phenotypic parameters of fruit tree branches, especially vine-like ones, and also provide necessary perceptual technology support for intelligent agricultural equipment.

## Data Availability

All data used to train and test the model presented in this study could be downloaded freely from https://github.com/abyssbjc/Reconstruction-of-passion-fruit-tree-branches.
